# Predicting microbial activity potential in salt caverns based on brine chaotropicity analysis

**DOI:** 10.1038/s41598-026-40866-z

**Published:** 2026-02-23

**Authors:** Abduljelil Kedir, Kyle Mayers, Janiche Beeder, Silvan Hoth, Nicole Dopffel

**Affiliations:** 1 NORCE Research AS, Nygårdsgaten 112, Bergen, Norway; 2 Equinor ASA, Sandsliveien 90, Sandsli, Norway

**Keywords:** H₂ storage, salt caverns, chaotropic, kosmotropic, agar rheology, brine composition, Environmental sciences, Microbiology

## Abstract

Salt caverns are promising sites for hydrogen (H₂) storage, but microbial activity in these high-salinity environments poses risks, including H₂ consumption and subsequent toxic hydrogen-sulfide (H₂S) production by sulphate-reducing bacteria. While salinity influences microbial diversity, the role of chaotropicity, defined as a membrane-disrupting effect of salts like magnesium chloride (MgCl_2_) and calcium chloride (CaCl_2_), remains unexplored. We introduce a novel method using oscillatory rheology to measure solute-induced changes in agar gel point temperature, enabling prediction of chaotropicity and subsequent microbial activity. We assessed individual salts, salt mixtures, literature data, and original brine samples from four salt caverns. Our results show that chaotropic conditions arise when the ionic strength (I) of solution exceeds 3 mol/L with 55% MgCl₂, or 6 mol/L with 40% MgCl₂. One tested cavern exhibited chaotropic properties, suggesting reduced microbial risk. Microbial analysis and growth tests confirmed missing microbial activity and minimal cell numbers in the chaotropic cavern, in contrast to the more kosmotropic caverns. Therefore, we propose a strategy to mitigate microbial threats by adjusting salt cavern brine composition to induce chaotropicity as one additional factor to limit activity, which offers a new framework for microbial risk management.

**Supplementary Information:**

The online version contains supplementary material available at 10.1038/s41598-026-40866-z.

## Introduction

As the world transitions towards cleaner energy sources, hydrogen (H₂) has emerged as a versatile and promising energy carrier. One of the key challenges in utilizing H₂ effectively is finding efficient and scalable storage solutions. Salt caverns have emerged as a promising option, drawing inspiration from the successful natural gas storage practices that began in the 1940s^1^. Salt caverns are created through a process known as solution mining, where fresh or seawater is used to dissolve underground salt deposits, forming large cavities. These caverns can reach volumes of up to 1 million cubic meters and can withstand pressures up to 300 bar, making them suitable for storing significant volumes of H₂^2,3^. The ability to rapidly inject and withdraw H₂ from these caverns provides a flexible solution for balancing energy supply and demand^4,5^. Despite their advantages, the use of salt caverns for H₂ storage is not without challenges. The presence of microorganisms in these high-salinity subsurface environments could pose a significant risk, as these microbes can utilize stored H₂ as an electron donor, leading to its reduction and possibly producing H₂S gas^2^. It was previously described that some salt caverns host diverse halophilic microbial communities, including sulphate-reducing bacteria, capable of surviving in high-salinity, low-nutrient conditions relevant to underground H₂ storage systems^2,6–8^. Mitigation and treatment of these high-salinity environments is challenging. Traditionally biocides are often employed to mitigate the adverse effects of microbial activity and associated risks. However, this conventional approach carries several challenges, including chemical degradation due to reactions within the high-salt brine, adsorption onto insoluble in the sump or other solid minerals, reducing their effectiveness. Also, these toxic compounds harbour general environmental- and health risks. Therefore, it is necessary to seek alternative methods to control or eliminate microbial activity.

Various physical and chemical factors can either limit or preserve microbial activity, which will in turn stabilize stored H₂^2^. One critical chemical factor influencing microbial survival is hypersalinity, which has been extensively studied to understand the limits of life in both terrestrial and Martian analogue environments. Understanding the physicochemical limits of microbial life is essential for assessing habitability in extreme environments^9–15^. While direct microbiological testing remains the gold standard, it is often time-consuming and labour-intensive. As an alternative and complementary approach, researchers have employed physicochemical parameters such as water activity (a_w_) and brine chaotropicity/kosmotropicity to define habitability thresholds. Water activity is defined as the availability of free water for biological processes, while chaotropicity and kosmotropicity describe the ability of solutes to disrupt or stabilize biological macromolecules, respectively^16^. Chaotropic agents weaken non-covalent interactions, destabilizing proteins and nucleic acids, whereas kosmotropic agents enhance water structure and stabilize macromolecules. These properties are influenced by both primary solutes (e.g., salts) and secondary solutes (e.g., macromolecules), which can interact in complex ways. Unlike water activity, chaotropicity provides a more comprehensive measure of the cumulative effects of solutes on macromolecular stability^17^.

Chaotropic and kosmotropic properties of a solution are determined using an agar gelling assay, in which solutes were evaluated based on their ability to decrease or increase the agar gelation temperature, respectively. In this assay, agar serves as a model macromolecular system that enables inference of solute-induced destabilization or stabilization effects.in^16,18–20^. Agar is a polymer that is made from a mixture of two galactose-based polymers: agarose and agar pectin, see Supplementary Fig. 1. The method involves measuring the temperature at which agar transitions from liquid to gel in the presence of various solutes, with chaotropic agents lowering and kosmotropic agents raising the gel point temperature. By calculating the energy change associated with a 1 °C shift in gelation temperature, the chaotropic or kosmotropic activity of each solute is expressed in kJ kg¹ mol¹.

While a universal scale for chaotropicity and kosmotropicity has been developed by Cray et al. (2013), it has not been applied to complex salt mixtures such as those found in salt caverns^20^. For example, sodium chloride (NaCl) is classified as a kosmotropic salt^9^, whereas magnesium chloride (MgCl_2_) is chaotropic^19,20^. MgCl_2_ has been used to define the upper chaotropicity limits for microbial life at a concentration of 2.30 M (i.e., I of 6.9 mol/L) in the absence of kosmotropic solutes^19^. On the contrary, Steinle et al. (2018) demonstrated active microbial life in the Kryos Basin with near-saturated MgCl_2_ (a_w_ ~0.4 and ionic strength of 11.47 mol/kg) previously thought to be uninhabitable^21^. Similarly, Yakimov et al. (2015) recovered mRNA from the Kryos interface layer with MgCl₂ concentrations up to 3.03 M, corresponding to a water activity of 0.631 and an ionic strength (I) of 9.1 mol/L, indicating recent gene expression and thus potential for active metabolic activity^22^. Gómez et al. (2019) report positive evidence of ultra-small life forms in the hyperacidic and hypersaline environment of Dallol volcano, Northern Afar, Ethiopia^23^. However, Moors et al. (2023) found no conclusive signs of active life in similar brines, attributing most DNA signals to contamination and emphasizing the role of chaotropicity and water activity as life-limiting factors. Belilla et al. (2022) further emphasized the need for strict controls in life detection, showing that although airborne microbes can reach the sampling area, they are rapidly destroyed by the brines, while mineral particles can mimic cells and bind fluorescent probes, leading to false positive results^24^. This highlights the complexity of defining the limits of microbial life in real-world conditions.

This study aims to assess the microbial risk potential for H₂ storage in artificial salt caverns using a newly developed approach of the agar gelling assay, with a particular focus on the role of inorganic salts commonly present in cavern brines. To date, the chaotropicity of salt cavern brines and their impact on microbial activity remain largely unexplored. Previous studies investigated the combined effects of chaotropic and kosmotropic salts exclusively using molar concentrations^25^. In contrast, we utilize molar ionic strength to predict the agar gelling temperature, allowing for a better comparison of the effects of monovalent and divalent ions regardless of their charge number. This method not only enhances our understanding of microbial viability in salt caverns or other extreme environments but also provides a framework for evaluating brine composition as a predictive tool. Our findings also offer alternative and practical recommendations for mitigating microbial risks, particularly during the early phases of screening and developing of salt caverns for H₂ storage.

## Methods

### Materials and chemicals

Agar used in this study was obtained from Difco™ Noble Agar and served as the gelling agent for determining the agar gelling point temperature. The chemical structure of agar, comprising agarose and agaropectin, is illustrated in Supplementary Fig. 1. Ultrapure water (18 MΩ·cm), produced using a Milli-Q system (Millipore), was used as the solvent for all preparations. The inorganic salts used in the experiments included sodium chloride (NaCl), potassium chloride (KCl), sodium sulfate (Na₂SO₄), magnesium chloride hexahydrate (MgCl₂·6 H₂O), calcium chloride dihydrate (CaCl₂·2 H₂O), and trisodium citrate (Na₃C₆H₅O₇, abbreviated as Na_3_Cit). All salts were of analytical grade (Merck). In addition, we obtained salt cavern samples (cavern 1–4) from three distinct localities in three countries in Europe, to analyze their microbial and chaotropic properties. Liquid brine samples (2 × 1 L) were taken either at the wellhead from the cavern brine (cavern 1 and cavern 2) or via downhole sampling from the cavern brine in the sump (cavern 3 and 4). Estimated cavern temperature for all caverns is between 40 and 45 °C. All liquid samples were taken anoxically and were transferred under constant nitrogen flushing into sterile glass bottles.

### Sample Preparation

Agar solutions at a concentration of 1.5 wt% (w/w) were prepared using either Milli-Q water or salt solutions. To prepare the solutions, the appropriate volume of Milli-Q water or salt solution was first weighed into flasks containing a magnetic stir bar. The required amount of agar powder was then added, and the flasks were sealed with lids. The mixtures were initially heated in a microwave until they approached boiling temperature, ensuring complete dissolution of the agar. Following this, the solutions were transferred to a magnetic stirrer set to 90 °C and stirred at 300 rpm. The mixtures were maintained under these conditions for at least one hour to ensure homogeneity before sampling for rheological measurements. Stepwise dilution was performed for samples with high salinity to prevent agar precipitation. The gel point temperatures of these diluted samples were subsequently estimated by extrapolation of the given function.

### Measurements

#### Rheology

A Paar Physica MCR 302 rheometer, equipped with a Peltier temperature-controlled hood (H-PTD 200) and integrated TruTemp™ and TruGap™ systems, was used to determine the gelling point temperature of agar solutions. Measurements were conducted using a concentric cylinder (CC27) geometry at zero gap to evaluate the viscoelastic properties of the samples. A temperature sweep test was performed from 70 °C down to either 20 °C–10 °C, depending on the expected gelling temperature, at a controlled cooling rate of 1 °C/min. In these tests, the shear strain was kept at 0.1%, and the angular frequency was set to 1 Hz, based on the results of preliminary amplitude sweep and frequency sweep tests. To ensure thermal stability, the measurement cup was preheated to the starting temperature (either 50–70 °C, depending on gelling temperature) for approximately 10 min before sample loading. After pouring the sample, it was allowed to equilibrate for 15 min before initiating the measurement. A two-piece lid was used to cover the cup to minimize evaporation during the test. The gelling point temperature was determined from the temperature-dependent profile of complex viscosity (η*). Most samples were measured in duplicate to assess the reproducibility of the results, which showed very good agreement between measurements on the onset of gelling point temperature, for example, see Supplementary Fig. 4. Data acquisition and analysis were performed using RheoCompass™ software.

#### Water activity

Water activity (a_w_) was measured using a Rotronic HC2-AW-USB probe connected to a PC via a USB interface. Samples were placed in WP-40TH sample holders equipped with a water jacket, which was connected to a thermostatically controlled water bath to maintain a constant temperature of 25.2 °C during the measurement. The instrument was calibrated before measurements using certified Rotronic calibration standards (EA10-SCS, EA35-SCS, and EA80-SCS) to ensure accuracy.

#### pH

The pH of the samples was measured using a HORIBA LAQUAtwin compact water quality meter. The probe was calibrated using a two-point calibration with standard buffer solutions at pH 4.01 and 7.00 before each measurement. Measurements were performed at 22 °C. For each sample, three parallel measurements were conducted, and the average value was reported.

#### Total salinity

The salinity of all samples was measured using an ATAGO PAL-SALT PROBE, which operates based on electrical conductivity. Before each measurement, the device was calibrated using a standard sodium chloride solution (e.g., 1% NaCl) to ensure accuracy. The instrument is capable of measuring salt concentrations within a range of 0.00% to 7.00%. Measurements were performed at 22 °C. Therefore, samples with salinity levels exceeding this range were subjected to stepwise dilution to reach the measurable limits of the device.

#### Composition analysis of salt cavern brines

Elemental analysis of inorganic cations was performed using an inductively coupled plasma optical emission spectrometer (ICP-OES), Optima 8300 DV (PerkinElmer, USA), following the standard operating procedure PT-TM-10.0155.03 E. The instrument is equipped with a dual-view plasma observation system, enabling both axial and radial detection to optimize sensitivity and accuracy across a broad concentration range. Calibration was conducted using certified multi-element standards, and quality assurance was maintained through the inclusion of blanks, duplicates, and certified reference materials to ensure compliance with the method and analytical reliability.

Inorganic anions were quantified using ion chromatography (IC) on a Dionex ICS 5000 + system (Thermo Fisher Scientific), following the procedure outlined in PT-TM-10.0250 E: *Determination of Anions in Water by Ion Chromatography*. The system was equipped with a Dionex IonPac AS11-HC analytical column (4 μm, 2 × 250 mm) and a corresponding AG11-HC guard column (4 μm, 2 × 50 mm), operated with a Dionex ECG 500 KOH eluent generator and an Anion ERS 500 suppressor in electrolytic mode. The eluent was degassed using an RFIC+ eluent degasser, and sample injection was performed via an ICS 5000 + AS autosampler. Before analysis, samples were filtered through 0.2 μm filter cartridges and transferred into 1.5 mL vials sealed with split-septum caps. A 25 µL injection volume was used, and the column temperature was maintained at 30 °C. Calibration was performed using certified multi-analyte standards, and quality control included blanks, duplicates, and check standards. Data acquisition and processing were conducted using Chromeleon™ Chromatography Data System software.

### Microbial analysis

#### Cell numbers

For DNA analysis, 20 mL of brine was filtered over a 0.22 μm filter directly on site after retrieving the liquid samples. Three filters per cavern were prepared this way and directly frozen for stabilization. DNA was extracted from the filters using the DNeasy Power Soil Kit (Qiagen). DNA concentration was determined via Qubit dsDNA High Sensitivity assay (Invitrogen). Copy numbers were measured via digital droplet PCR (ddPCR; BioRad) using specific 16 S rRNA Bacteria primer^26^. The ddPCR reactions were run with a total volume of 20 µL on a DX200 instrument (BioRad) using 1x EvaGreen supermix (BioRad) and 250 nM (final concentration) of primers. Complete PCR reactions were emulsified with QX200 Droplet Generation Oil for EvaGreen using the QX200 Droplet Generator and then transferred to a 96-well plate. PCR reactions were performed in a C1000 Touch Thermocycler with deep-well module (BioRad) using the following program: 95 °C for 15 min, 40 cycles of 95 °C for 30 s, 61 °C for 1 min, 4 °C for 5 min, 90 °C for 10 min and finally an infinite hold at 4 °C. Plates were equilibrated to room temperature for at least 10 min before being analysed on a QX200 Droplet Reader (BioRad). Copy numbers were calculated to cell numbers using assumed number of genes: 5.3 copies/cell for Bacteria (based on the rrnDatabase). Copy numbers were corrected regarding the non-template control. To rule out inhibition of the PCR reaction by the salt, we included a positive control of bacterial DNA (*E.coli*) to check the amplitude of the positive droplets from salt cavern samples in comparison to the positive control. We did not see reduced fluorescence amplitude of positive droplets, so PCR inhibition can be ruled out.

#### Cultivation tests

An important fact is that salt caverns are anoxic. After the initial solution-mining phase there is no input of oxygen. A good indicator is dissolved iron (II) in the brines, which oxidize quickly when exposed to air. Therefore, we employed anaerobic cultivation tests to investigate microbial activity. For this 25 mL of pure brine was filled in individual sterile bottles with different gas mixtures in the headspace. No artificial media was used. The bottles were closed with butyl rubber stoppers. We tested for different microbial activity including fermentation and H₂-consumption. To test for fermentation, which is a fast metabolic process, we added the nutrients 10 mM glucose + 0.01% yeast extract. To test for H₂ consumption, which can be a slow process under high-salt conditions, we added 100% H_2_ in the headspace in addition to 10 mM acetate + 0.01% yeast extract as nutrient boost. Controls included bottles with anoxic, distilled water with 100% H_2_ to measure H_2_ leakage through stoppers. Activity was confirmed via gas- or liquid products, pH increase, and H₂ consumption over time. Incubation was done at different temperatures, including the cavern temperatures (between 40 °C and 45 °C). Each sample was incubated and regularly analysed for at least one year or longer.

## Results and discussion

### Method for chaotropicity and kosmotropicity measure

The gelling temperature of agar has been previously determined either by visual inspection in conjunction with thermometer-based temperature monitoring^16^ or through temperature-resolved UV-visible absorption spectroscopy^18^. Here, we propose a new method that utilizes a rheometer to evaluate solute-induced shifts in the gelling point temperature of agar, to determine the chaotropic or kosmotropic behavior of inorganic salts. Both rotational and oscillatory rheological techniques can be employed for this purpose; however, oscillatory rheology is preferred due to its ability to provide detailed insights into the viscoelastic properties of materials at equilibrium and over short time scales. During method development, various geometries were assessed to identify the most suitable configuration for measuring the gelling point of agar. Initial trials using parallel plate geometries (25 mm and 50 mm diameters) and a cone-plate system (50 mm diameter), combined with a Peltier temperature-controlled hood (H-PTD 200) and a solvent trap, revealed limitations: high salinity in the solvent led to edge drying, resulting in artificially elevated viscosity readings. Additionally, sedimentation in these configurations could cause an underestimation of viscosity, as only the supernatant phase would be measured. To overcome these issues, we adopted a concentric cylinder (CC27) geometry, which minimizes the effects of surface drying and sedimentation. Although this setup requires a larger sample volume and does not support the TruTemp™ feature, potentially introducing slight temperature gradients, we mitigated this by preheating the system to the target temperature for 10 min and allowing the sample to equilibrate for 15 min before measurement.

**Fig. 1:**
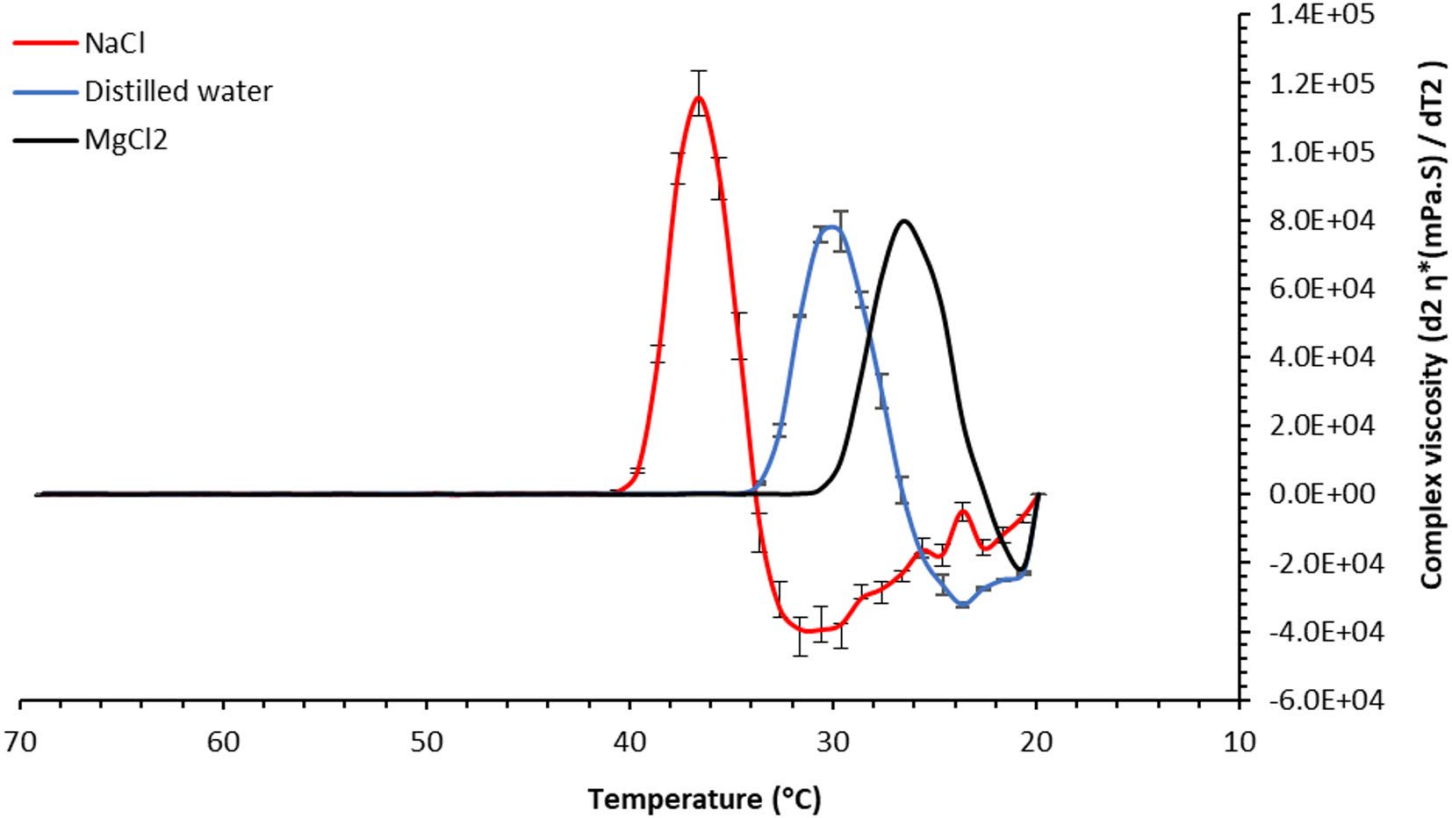
Examples of second derivative of viscosity versus temperature: *Sweep measurement of 1.5% agar in distilled water (blue)*,* NaCl (red) (I of 2.18 mol/L or 2.18 Molarity)*,* and MgCl*_*2*_ *(black) (I of 2.18 mol/L or 0.73 Molarity)*.

To define the linear viscoelastic region, the strain sweep test was conducted over a shear strain range of 0.01% to 100% at an angular frequency of 10 Hz and a temperature of 50 °C (see Supplementary Fig. 2). Frequency sweep tests were then performed across a range of 0.1 to 100 Hz at a constant shear strain of 0.1% and the same temperature to identify the elastic-dominant frequency range (see Supplementary Fig. 3). Both tests were conducted using agar solutions prepared in distilled water, and the resulting data were used to inform the parameters for subsequent temperature sweep experiments. Finally, a temperature sweep test was performed, varying from 70 °C to either 20 °C–10 °C, depending on the sample type, at a rate of 1 °C/min while setting shear strain at 0.1% and angular frequency at 1 Hz (see Supplementary Fig. 4). Each measurement lasted 75 to 85 min. The onset of the agar gelling point temperature was estimated from the plot of complex viscosity (η*, calculated from the storage modulus (G’) and loss modulus (G’’)) as a function of temperature. We analyzed the second derivative of the complex viscosity versus temperature plot to highlight the smooth and distinct transition from a liquid-like state to the gelling of the agar samples (Fig. 1). Several samples were measured in duplicate to assess the uncertainty of the measurements on the onset of gelling temperature, with standard deviations.

The influence of pH on agar gelation was investigated by Hallsworth et al. (2003), who reported that the gelling temperature of agar remains stable across a pH range of pH 2 to pH 11^16^. Therefore, any observed changes in gelling temperature in this study can be attributed to the solute’s chaotropic or kosmotropic effects, rather than pH-induced alterations. This ensures that the assay provides a reliable, pH-independent measure of solute-induced macromolecular destabilization or stabilization. In previous studies, the classification of salts as chaotropic or kosmotropic has often been based on their influence on the gelling point temperature of agar. Specifically, if the onset of the gelling temperature in the presence of a given salt is higher than that observed in distilled water, the salt is considered kosmotropic, resulting in a negative value. Conversely, a lower gelling temperature indicates chaotropic behaviour, which results in a positive value. Kosmotropic activity has been quantified in kJ/kg·mol, using the specific heat capacity of agar in distilled water (4.15 kJ/g) to estimate the energy change associated with each 1 °C shift in gelling temperature. However, we suggest a simplified yet conceptually consistent approach by reporting chaotropic and kosmotropic activity directly as the change in gelling temperature (ΔT), rather than converting it to energy units. This decision is justified by the fact that all samples were prepared at a fixed ionic strength, and the conversion to energy units would not significantly alter the interpretation of the results. The change in gelling temperature is calculated using Eq. 1:

1 $$\:\varDelta\:\boldsymbol{T}={\boldsymbol{T}}_{2}-{\boldsymbol{T}}_{1}$$

Where T_2_ ​ is the onset gelling temperature in the presence of salt, and T_1_ is the reference gelling temperature in distilled water. In this framework, a positive ΔT indicates kosmotropic activity, while a negative ΔT signifies chaotropic behaviour. For example, the onset gelling temperatures were measured as 40.6 °C for NaCl, 34.6 °C for distilled water, and 31.6 °C for MgCl₂ (see Fig. 1). Based on Eq. 1, NaCl exhibits a + 6 °C shift, indicating kosmotropic behaviour, whereas MgCl₂ shows a − 3 °C shift, consistent with chaotropic activity. Although the magnitude of the temperature change remains consistent with previous interpretations, our approach reverses the direction of the temperature change. This adjustment represents the intrinsic nature of chaotropic and kosmotropic effects, aligning the sign with the solute’s influence on the gelling point temperature. To summarize, the new method—based on rheological measurements—offers an alternative approach for evaluating the chaotropic and kosmotropic properties of salts. Furthermore, beyond assessing solute effects on gel formation, this approach also provides insights into the viscoelastic behaviour of agar under different chemical environments, offering indirect evidence of the chemical stresses exerted on its macromolecular structure.

### Chaotropic and kosmotropic effects of a single salt

This test aims to investigate how individual salts influence the gelation temperature of agar. Notably, Cray et al. (2013) conducted extensive work establishing a universal scale of chaotropic and kosmotropic behavior based on a comprehensive screening of 97 organic and inorganic compounds^18^. In contrast, our study focuses specifically on inorganic salts commonly found in terrestrial environments, particularly those associated with salt caverns. To ensure accurate comparisons between monovalent and multivalent ions, we used the ionic strength of the solution rather than molar concentration. Ionic strength accounts for differences in ion charge, providing a more meaningful basis for evaluating chaotropic and kosmotropic effects. Some previous studies have relied on molar concentration (e.g., molarity) to directly compare chaotropicity of salts such as magnesium sulfate (MgSO_4_)and MgCl_2_, despite differing ionic strengths, which may lead to misinterpretations of the results^27^. Therefore, our approach standardizes comparisons by maintaining a consistent ionic strength across all test conditions, whether using single salts or mixtures.

**Fig. 2:**
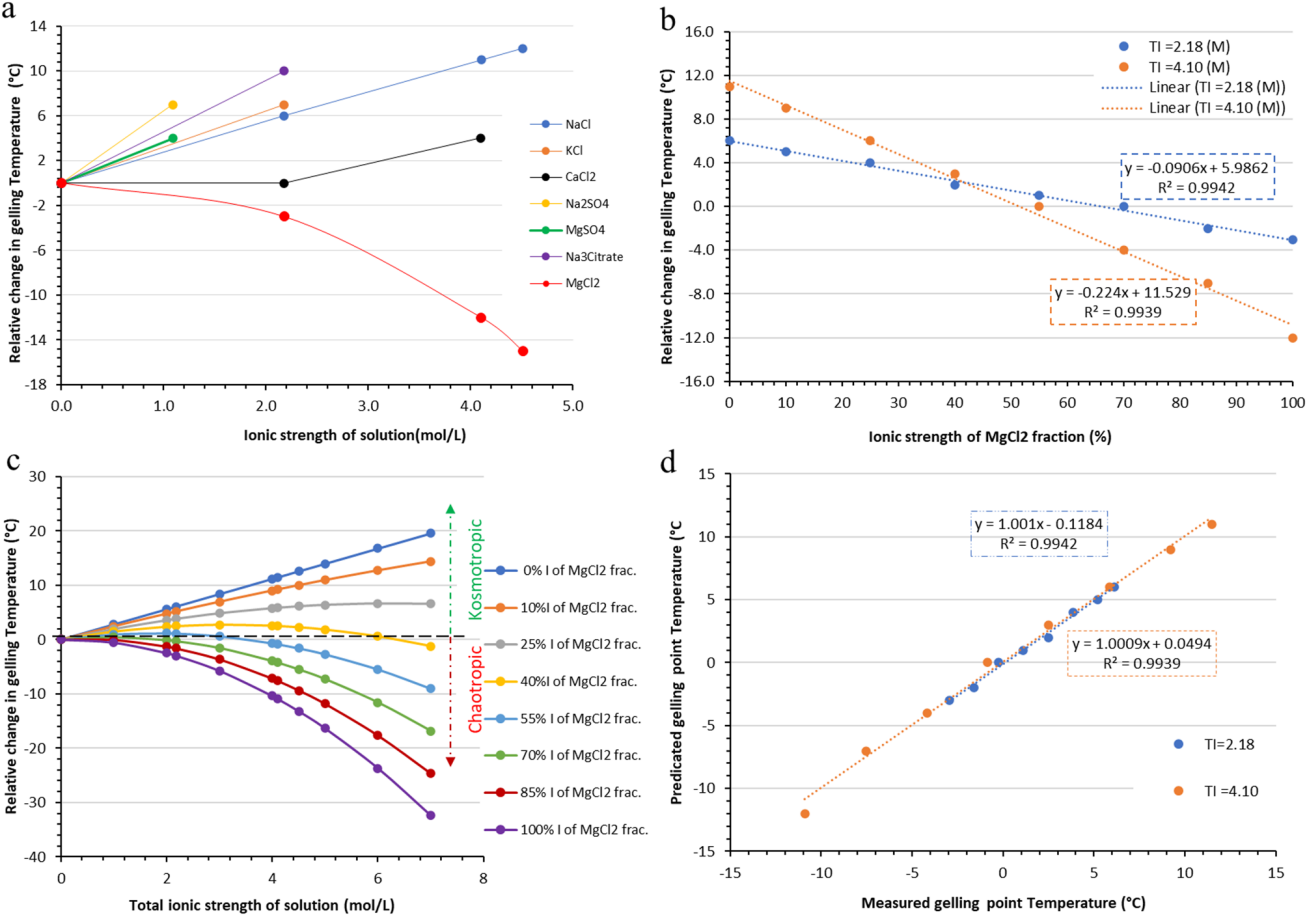
Detailed investigation of change in gelling temperature of kosmotropic and chaotropic salt: **a**). The relative change in agar gelling point temperature of 1.5wt % agar in NaCl, KCl, CaCl_2_, MgCl_2_, Na_2_SO_4_, MgSO_4_, and Na_3_Cit as a function of their ionic strength of solution. (**b**) The measured (M) relative change in gelling point temperature of 1.5wt % agar as a function of ionic strength fraction of MgCl_2_ while maintaining the total ionic strength of the solution at 2.18 and 4.10 mol/L. (**c**). Predicted relative change in gelling point temperature of 1.5wt % agar in a mixture of MgCl_2_ and NaCl as a function of the total ionic strength of the solution while varying the ionic strength fraction of MgCl_2_. (**d**). A comparison between measured (M) and predicted (P) relative change in gelling point temperature of 1.5wt % agar in a mixture of MgCl_2_ and NaCl as a function of the total ionic strength of solution at 2.18 and 4.10 mol/L while varying the ionic strength fraction of MgCl_2_.

To ensure biological relevance, we initially based our test conditions on the brine composition of growth media used for halophilic microorganisms, such as the *Desulfohalobium* (DH) medium^28^. This medium supports robust microbial activity, thereby validating the applicability of the proposed agar method. The DH medium primarily contains inorganic salts, namely, chlorides of sodium, magnesium, and calcium, along with sulfates, which collectively contribute to a calculated ionic strength of 2.18 mol/L. These salts account for approximately 99% of the total ionic strength (TI) of the medium. To investigate the specific effects of individual salts, we systematically replaced the complex salt mixture with single inorganic salts, adjusting their concentrations to maintain a constant ionic strength equivalent to that of the original DH medium. Table 1 lists the salts tested and their corresponding effects on agar gelation temperature, which serve as indicators of their chaotropic or kosmotropic character. In addition, Fig. 2a illustrates the relative changes in the agar gelling point temperature of 1.5 wt% agar in NaCl, KCl, CaCl_2_, MgCl_2_, Na_2_SO_4_, MgSO_4_, and Na_3_Cit as a function of their ionic strengths. The results reveal that the relative agar gelation temperatures in the presence of NaCl, KCl, Na₂SO₄, MgSO₄, and Na₃Cit exhibit a positive linear correlation with ionic strength, except CaCl₂, indicating the kosmotropic behavior. In contrast, MgCl₂ displays a negative polynomial relationship with ionic strength, classifying it as a chaotropic salt. To better characterize these trends, additional concentration points for NaCl, CaCl₂, and MgCl₂ were included beyond those listed in Table 1. Moreover, the concentration of Na_2_SO_4_ and MgSO_4_ was diluted by half due to significant precipitation of Na_2_SO_4_ and mild precipitation of MgSO_4_ at an ionic strength of 2.18 mol/L.

**Table 1:** List of inorganic/organic salts used to measure the chaotropic and kosmotropic effect and the calculated amount of molar ionic strength of solution at which there is a 1 °C increase (+) or decrease (-) in agar gel-point, including data taken from Cray et al. (2013)^18^, which is recalculated from their molar concentration.

Type of salt	Molarity of solution (mol/L)	Molar ionic strength of solution (mol/L)	Molar ionic strength of solution at which there is a 1 °C increase (+) or decrease (-) in agar gel-point (mol/L) measured in this study	Molar ionic strength of solution at which there is a 1 °C increase (+) or decrease (-) in agar gel-point (mol/L) data taken from Cray et al. (2013). ^18^
NaCl	2.18	2.18	0.37	0.38
KCl	2.18	2.18	0.31	0.37
CaCl_2_. 2 H₂O	0.73	2.18	2.70	0.14
MgCl_2_. 6 H₂O	0.73	2.18	0.70	0.23
MgSO_4_. 7 H₂O	0.54	2.18	0.26	0.26
Na_2_SO_4_	0.73	2.18	0.16	0.25
Na_3_Cit	0.36	2.18	0.22	0.39

Interestingly, while the chaotropicity of CaCl_2_ is typically assessed using gelatin, due to its tendency to precipitate agar, this experiment evaluated CaCl_2_’s chaotropicity in agar without any observed precipitation. This unexpected result indicated that CaCl_2_ acted more like a kosmotropic salt rather than a chaotropic salt, contradicting previous reports^18^. Though earlier studies have compared the chaotropicity of CaCl_2_ measured in gelatine, assessment in agar might be questionable given the fundamental differences in chemical structure and properties between agar and gelatine. Besides, calcium-rich brines are relatively rare in natural environments due to the low solubility of calcium carbonate and calcium sulphate, particularly in the presence of carbonate and sulfate ions^29^. This geochemical constraint reduces the relevance of CaCl₂ in evaluating brine chaotropicity, especially in salt caverns dominated by halite and carnallite. For our tests we therefore selected salts that are abundant in the terrestrial environment, including those commonly found in salt caverns (see Supplementary table S2). The considered concentration range spans from zero to saturation, which our model is designed to predict, with salt cavern samples serving as a representative example.

Cray et al. (2013) previously reported the concentrations (in mM) at which various inorganic salts caused a ± 1 °C shift in agar gelation temperature. The corresponding ionic strengths were calculated and are presented in Table 1. In this study, we also estimated the ionic strength required to induce a 1 °C change in gelation temperature using curve fitting. The calculated values for NaCl, KCl, Na₂SO₄, and MgSO₄ showed strong agreement with Cray et al. (2013) ‘s findings. However, differences were observed for Na₃Cit, CaCl₂, and MgCl₂, which may be attributed to differences in agar type resulting in potential selective interactions with the different salts. The key difference lies in the concentration required to induce a 1°C change in gelation temperature compared to the findings of Cray et al. (2013). We employed high-quality agar (Nobel agar), which differs from the extra-pure reagent-grade agar (Nacalai Tesque, Kyoto, Japan) used in earlier studies. This distinction in material quality may also contribute to the observed differences in gelation behaviour. Still, the overall interpretation remains consistent, aside from differences in magnitude.

### Chaotropic and kosmotropic effects of binary salt mixtures

We further investigated the influence of salt mixtures on the gelation temperature of agar, with a focus on distinguishing their chaotropic and kosmotropic characteristics. The primary objective was to simulate the ionic composition of salt cavern brines using a binary salt system. Salt caverns, typically rich in halite and carnallite, yield brines predominantly composed of sodium chloride (NaCl) and magnesium chloride (MgCl₂), with minor contributions from other ions. To represent this geochemical environment, a binary mixture of NaCl (a kosmotropic salt) and MgCl₂ (a chaotropic salt) was selected. Thus, binary solutions were prepared with varying ionic strength fractions I of NaCl and MgCl₂, while maintaining a constant total ionic strength (TI) of either 2.18–4.10 mol/L. These TI values were chosen to align with previous measurements and to ensure that the system exceeded the threshold at which a ± 1 °C shift in agar gelation temperature was observed for individual salts. Table 2 presents the concentrations and corresponding ionic strengths of NaCl–MgCl₂ mixtures at a TI of 2.18 mol/L, with varying I of MgCl₂ frac. A similar dataset for TI = 4.10 mol/L is provided in Supplementary Table S1. This design enables a systematic evaluation of how the balance between kosmotropic and chaotropic components influences the gelation behavior of agar.

**Table 2:** The concentration and the corresponding ionic strength of binary salt mixtures of NaCl and MgCl_2_ with varying I of MgCl_2_ frac., while maintaining the total ionic strength (TI) at 2.18 mol/L.

[NaCl] (mol/L)	[MgCl_2_.6 H₂O] (mol/L)	I of NaCl	I of MgCl_2_.6 H₂O	I of MgCl2 fraction (%)	TI (mol/L)
2.18	0.00	2.18	0.00	0%	2.18
1.95	0.08	1.95	0.23	10%	2.18
1.63	0.18	1.63	0.55	25%	2.18
1.30	0.29	1.30	0.87	40%	2.18
0.98	0.40	0.98	1.20	55%	2.18
0.65	0.51	0.65	1.53	70%	2.18
0.33	0.62	0.33	1.85	85%	2.18
0.00	0.73	0.00	2.18	100%	2.18

The gelation temperature of 1.5 wt% agar was measured in binary salt mixtures of NaCl and MgCl₂ at two total ionic strengths (TI): 2.18 mol/L and 4.10 mol/L. Figure 2b shows the measured (M) relative change in gelation temperature as a function of I of MgCl₂ fraction. At both TI levels, the results reveal a clear negative linear relationship between the relative gelation temperature and increasing I of MgCl₂ frac. This trend indicates a progressive shift in behavior from kosmotropic to chaotropic as the proportion of MgCl₂ increases in the mixture. Notably, the slope of the linear trend becomes steeper at the higher TI (4.10 mol/L), suggesting that a lower I of MgCl₂ frac. is required to induce chaotropic behavior at elevated ionic strengths. Linear regression analysis estimates the onset of chaotropicity, defined as the I of MgCl₂ frac. at which the gelation temperature begins to decrease, at approximately 66% for TI = 2.18 mol/L and 51% for TI = 4.10 mol/L. These findings support the hypothesis that the balance between kosmotropic and chaotropic components in brine significantly influences the structural integrity of agar gels.

To extend the analysis, a predictive model was developed to estimate the relative change in the gelling-point temperature of 1.5 wt% agar as a function of both the ionic strength of MgCl₂ fraction and the total ionic strength (TI) of the solution. This was achieved through a two-step approach: first, predicting the agar gelling-point temperature of salt mixtures as a function of TI, and second, using the first prediction to estimate the agar gelling-point temperature as a function of ISF of MgCl_2_. Both steps are described below in detail. In the first step, we separately collected the slopes and y-intercepts of 1.5 wt% agar gelling-point temperature at every point of TI, i.e., 2.18 and 4.10 mol/L. In addition, at 0 mol/L TI, both the slope and y-intercept are zero, serving as a baseline and introducing a third point into the new function. Hence, one can create a new function using these three TI points and the corresponding slope point. Similarly, the same can be done using the y-intercept points. Subsequently, Supplementary Fig. 5 shows the function of the slope and y-intercept of 1.5% agar gelling point temperature in a mixture of NaCl and MgCl_2_ as a function of the total ionic strength of the solution (TI). In the second step, the functions from the first step were applied to estimate the gelling-point temperature as a function of the I of MgCl₂ frac., enabling the generation of a continuous prediction surface that captures the combined effects of TI and ISF on agar gelation behaviour. This new function aids in predicting a function, similar to what is seen in Fig. 2b, for any other TI. In addition, Supplementary Fig. 5 illustrates that the slope of the gelling-point temperature function increases with TI in a manner consistent with the chaotropic behaviour of MgCl₂, while the y-intercept follows a trend similar to NaCl, reflecting its kosmotropic influence.

Using this model, a comprehensive prediction was generated for the relative change in gelling-point temperature across a range of TI values and I of MgCl₂ frac. (see Fig. 2c). To validate the model, predicted values were compared with measured data at TI levels of 2.18 and 4.10 mol/L. The comparison (Fig. 2d) showed a strong linear correlation (*R* = 0.994), confirming the model’s accuracy. Additionally, the magnitude of temperature change increased with increasing TI at any given I of MgCl₂ fraction. Figure 2c further reveals that the chaotropic/kosmotropic behaviour of the binary salt mixture is governed by both TI and I of MgCl_2_ or NaC fractionl. Chaotropic effects are negligible below 0.5 mol/L TI, regardless of the Iof MgCl_2_ fraction.At higher TI, chaotropicity emerges only when the I of MgCl₂ fraction. exceeds 40%. A fully chaotropic environment requires a TI of at least 3 mol/L and an I of MgCl₂ fraction. above 55%. This indicates that both high ionic strength and a dominant chaotropic component are necessary to induce chaotropic behaviour in brine.

**Fig. 3:**
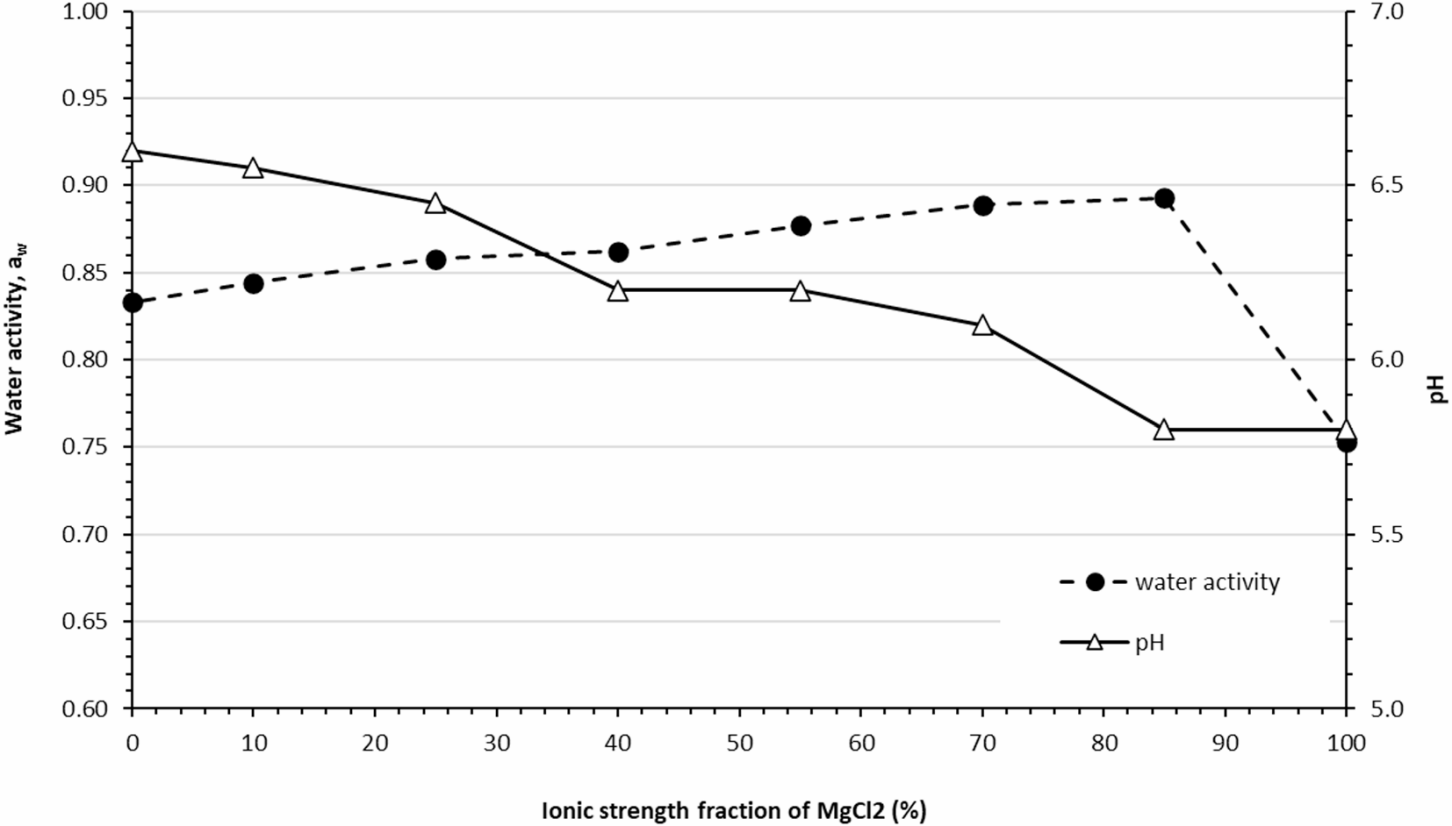
Water activity, a_w_ and pH of NaCl-MgCl_2_ mixtures: The a_w_ (● dotted line) and pH measurement (ρ solid line) of a binary mixture of MgCl_2_ and NaCl as a function of the ionic strength fraction of MgCl_2_ while maintaining the total ionic strength of the solution at 4.10 mol/L.

To complement the chaotropicity measurement, a_w_ and pH were measured for the binary mixture at TI of 4.10 mol/L. These parameters were selected due to their sensitivity to the high ionic strength of the solutions. Figure 3 shows the measured a_w_ and pH of the binary mixture of MgCl_2_ and NaCl as a function of the I frac. of MgCl_2_, while maintaining TI at 4.10 mol/L. The pH of the binary salt mixture decreases with increasing I of MgCl₂ fraction. This trend is expected, as MgCl₂ introduces a higher concentration of chloride ions compared to NaCl, and Mg²⁺ ions exhibit a stronger hydration capacity than Na⁺ ions, contributing to increased acidity in the solution. Interestingly, a_w_ initially increases as the I of MgCl₂ fraction. rises to approximately 85% but then decreases when the I fraction reaches 100%. As anticipated, the a_w_ of pure MgCl₂ is lower than that of NaCl at the same TI, due to the strong hydration and structure-disrupting effects of MgCl_2_, which interfere more significantly with H₂ bonding in water. Therefore, replacing NaCl with MgCl₂ at constant TI would theoretically result in a steady decline in a_w_. However, the observed results deviate from this expectation, showing an opposite trend in the mixed salt solutions, except at the endpoints of pure NaCl and pure MgCl₂. This anomaly suggests a possible hysteresis effect in the system, indicating that the behaviour of a_w_ in mixed salt environments may not follow a simple additive model. Further investigation is needed to understand the underlying mechanisms driving these deviations.

In addition, we attempted to predict the agar gelling-point temperature of NaCl–MgCl₂ mixtures using the individual response functions of NaCl and MgCl₂. However, this approach failed to accurately capture the behaviour of the binary system, except at the concentration extremes where additive effects dominate. In the mid-range, significant deviations were observed, suggesting non-additive interactions—possibly due to hysteresis or complex ion–water–polymer dynamics. These findings highlight the importance of directly testing salt mixtures to better understand their combined effects, which is essential for accurately predicting brine chaotropicity.

Initial results indicate that microbial activity limits in such environments are influenced not only by the total ionic strength (TI) of the solution but also by the ionic strength fraction of the chaotropic component, particularly MgCl₂. Importantly, this study introduces a predictive model that establishes the chaotropic threshold of brines in the presence of kosmotropic solutes, utilizing the agar gelling assay.

### Chaotropic and kosmotropic effects of high-salinity brines

To validate the predictive model, we compared its outputs with experimental measurements from salt cavern brine samples. Specifically, the chaotropic and kosmotropic characteristics of four salt cavern brine samples were assessed using the agar gelling assay. Caverns 1, 3 and 4 lie in different geological domains of the same salt basin, with caverns 3 and 4 belonging to the same cavern field. Cavern 2 in contrast, is located in a much younger, geographically distinct salt basin. To support the interpretation of these results, additional measurements of salinity, pH, and a_w_ were conducted (see Table 3). Salinity measurements confirmed that all four samples had reached the saturation level. The a_w_ data indicated that the first three samples closely resembled saturated NaCl solutions, suggesting NaCl dominance. In contrast, the fourth sample exhibited lower a_w_ than saturated NaCl, implying the presence of additional salts with stronger water-structuring effects. These observations were supported by ion composition analysis, performed using ICP-OES for cations and IC for anions. The detailed brine compositions are provided in Supplementary Table S2. Total dissolved solids (TDS) ranged from 325 to 337 g/L, and total ionic strength (TI) varied between 5.6 and 7.5 mol/L. Sample 1–3 was dominated by Na⁺, while sample 4 contained a more balanced mix of Na⁺, K⁺, and Mg²⁺. All samples exhibited high concentrations of Cl⁻, the dominant anion, consistent with the formation of chloride-based salts.

**Table 3:** The result of pH, a_w_, total ionic strength of solution (TI), ionic strength (I) of MgCl_2_ frac, and the measured relative agar gelling temperature of four cavern samples.

Samples	pH	Water activity, a_w_	TI (mol/L)	I MgCl_2_ frac.(%)	Predicted Gelling Temp (°C)	Measured Gelling Temp (°C)
Salt cavern 1	7.48	0.747	5.74	2.2	15	15
Salt cavern 2	6.93	0.751	5.63	0.2	16	15
Salt cavern 3	6.48	0.753	6.21	14.5	11	16
Salt cavern 4	5.4	0.693	7.53	57.9	-13	-3

A predictive assessment was conducted based on the total ionic strength (TI) and the ionic strength fraction of MgCl₂, indicating that salt cavern samples 1–3 were kosmotropic, while sample 4 exhibited chaotropic characteristics (see Table 3). To validate these predictions, the agar gelling-point temperature was experimentally measured for all four samples. However, due to the extremely high ionic strength of the brines, direct measurement was hindered by agar precipitation. To overcome this, a stepwise dilution approach was employed, using three dilution levels: 25%, 50%, and 75% (see Supplementary Fig. 6). The relative change in agar gelling-point temperature was determined by extrapolating from the diluted samples to estimate the behavior of the undiluted brines (Table 3). The results confirmed that samples 1–3 exhibited kosmotropic behavior, while sample 4 displayed chaotropic characteristics, consistent with the model’s qualitative predictions. However, while the classification of sample 4 as chaotropic aligned with expectations, a notable discrepancy was observed in the magnitude of chaotropicity between the predicted and measured values. This suggests that although the model effectively captures the general trend, additional factors may influence the quantitative response in complex brine systems, which need to be further investigated.

All brines were investigated regarding bacterial cell numbers (calculated from 16 S rRNA gene copy numbers) and occurring microbial activity when incubated under lab conditions. The obtained bacterial cell numbers reflect the overall trends across the site. Specifically, cavern 4 contains almost no bacterial cells (Table 4). We want to highlight that the calculated cell numbers are based on an average copy per cell. It is known that bacteria can have varying 16 S rRNA copy numbers, so cell numbers are only an estimate. Furthermore, we employed Bacteria-specific primer and did not further investigated Archaea. This should be done in future studies. To test general microbial activity potential, we tested for two metabolisms commonly present in anoxic environments: (a) fermentation on complex carbon sources, which is a metabolic trait in many microbes; and (b) H_2_-oxidation via anaerobic respiration like sulphate reduction. Fermentation is a quick process, resulting in visible microbial growth normally within days or weeks. Microbial H_2_-oxidation at high-salinity conditions, however, is a slow process and activity might take up to a year or longer^2^. Both tests aimed to show whether the general chaotropic behavior of the cavern aligns with microbial growth tests, which are normally needed to assess the microbial risk of a salt cavern. Indeed, cavern 4 did exhibit no microbial activity, even after over one year of incubation (Table 4). We assume that lack of general microbial activity is due to the combination of high chaotropicity, low pH, and low a_w_, which collectively render cavern 4 a low-risk cavern for H₂ storage. In contrast, the other three investigated caverns showed both significant microbial cell numbers and microbial fermentation activity (Table 4and Supplemental Table 3). Additionally, cavern 1–3 showed measurable H₂ consumption, also indicated by a pH increase (Table 4 and Supplemental Tables 4–7). Cavern 1 and 2 accumulated H_2_S in the headspace, a clear sign of ongoing sulphate-reduction. Of course, the activity in cavern 1–3 was boosted by nutrient addition and does not reflect the in-situ activity during H_2_ storage. Nevertheless, the results suggest that chaotropicity measurements can be related to microbial activity in general. One limiting aspect of these growth tests is the fact that many microbes are not culturable under lab conditions or incubations might take even longer than one year to observe activity.

**Table 4:** Microbial cell enumerations and activity indicators of the growth tests using pure salt cavern brine. Two types of anaerobic activity were tested: (a) fermentative activity on glucose and (b) H_2_ consumption.

	Cavern 1	Cavern 2	Cavern 3	Cavern 4
Cell numbers [cells/mL]	2.50E + 03	2.00E + 04	1.30E + 01	3.10E + 00
Incubation time to test fermentation	56 days	30 days	307 days	208 days
Glucose consumed [mg/L]	− 447	-1034	-94	no
Acetate produced [mg/L]	+ 136	no	+ 12	no
H_2_ produced?	yes	yes	yes	no
Incubation time to test H_2_ consumption	176 days	363 days	312 days	403 days
H_2_ consumed [mmol]	-0.19*	-0.08*	-0.10*	no
maximum H_2_S produced [ppm]	1561	1857	no	no
maximum pH increase	+ 1.1	+ 1.2	+ 0.3	+ 0.1

*corrected to abiotic H_2_ loss from water control bottles.

**Table 5:** The sample pH, water activity, predicted, measured, and calculated agar gelling point temperature from this study and data collected from the work of Payler et al. (2019), Moors et al. (2023), and Belilla et al. (2019 and 2021).

Samples	pH	a_w_	TI (mol/L)	I of MgCl_2_ frac.(%)	Predicted Gelling Temp (°C) of this study	Measured /Predicted Gelling Temp (°C) of published data	Microbial activity observed	Reference
Billingham	7.12	0.73	5.32	0.3	15	16	Yes	Payler et al. (2019)
215	7.25	0.71	5.38	0.2	15	16	Yes	
29XC	4.96	0.73	5.32	45	0	22	Yes	
44XC	5.15	0.74	5.55	28	6	18	Yes	
101-P	3.42	0.57	6.56	60	-10	-30	No	
Central green pool (1)*	0.7	0.73	5.56	32	3	5	No	Moors et al.(2023)
Left green pool (2)*	0.8	0.73	5.44	33	3	5	No	
Right yellow pool (3)*	0.9	0.73	5.63	28	5	6	No	
White chimney envi(4)*	0.7	0.73	5.29	29	5	5	No	
Gaet’ale Pond (5)	3.4	0.23	18.08	32	-36	-115	No	
Black Lake (6)	2.8	0.32	14.93	95	-141	-66	No	
Yellow mini pond (7)	3.7	0.68	9.31	35	-4	-36	No	
Red micro pond (8)	3.5	0.46	15.04	41	-38	-84	No	
Asale karst hole (9)	4.4	0.73	5.53	7	13	-2	Yes	
Karum Lake (10)	4.5	0.74	6.14	6	15	0	Yes	
DAL 4.00 (Dallol D)*	-0.45 ± 0.22	0.72	6.104	30	4.59	5.23	No	Belilla et al. (2019)
7DAL4-W1 (Dallol D)*	-0.45 ± 0.23	0.67	6.314	55	-6.51	-4.65	No	
7DAL4 W2(Dallol D)*	-0.45 ± 0.24	0.70	5.383	42	0.75	-2.00	No	
7DAL7 (Dallol D)*	-0.45 ± 0.25	0.69	5.989	50	-3.44	-2.12	No	
7DAL—N 1 (Dallol D)*	-0.45 ± 0.26	0.69	6.472	50	-4.77	-2.22	No	
7DAL—N2 (Dallol D)*	-0.45 ± 0.27	0.70	5.94	45	-1.52	-2.77	No	
7DAL9(Dallol D)*	-0.45 ± 0.28	0.71	5.176	33	3.81	1.98	No	
7DAL10 (Dallol D)*	-0.45 ± 0.29	0.71	5.037	31	4.57	-0.51	No	
7DAL12 (Dallol D)*	-0.45 ± 0.30	n.d	5.793	30	4.84	7.52	No	
7DAL13-W1(Dallol D)*	-0.45 ± 0.31	0.72	4.785	11	10.16	5.98	No	
7DAL14 (Dallol D)*	-0.45 ± 0.32	0.75	5.307	35	3.11	2.82	No	
BL (Black Lake)	3.04 ± 0.66	0.32	19.155	94	-229.14	-69.47	No	
7BL-W1 (Black Lake)	3.04 ± 0.67	0.32	14.206	95	-126.74	-47.83	No	
7BL-W2 (Black Lake)	3.04 ± 0.68	n.d	14.721	95	-136.13	-48.51	No	
7 Gt (Cave water)	6.36 ± 0.16	0.73	4.751	5	11.89	4.41	Yes	
8 Gt (Cave water)	6.36 ± 0.17	0.73	6.873	3	17.60	13.86	Yes	
7YL-W1 (Yellow Lake)	1.93 ± 0.44	0.26	18.446	28	-26.99	-77.30	No	
7YL-W2 (Yellow Lake)	1.93 ± 0.45	0.47	13.796	34	-17.12	-74.27	No	
8Ass (Lake Assale )	6.54	0.72	7.274	7	16.46	n.d	Yes	
PS3 (Lake Assale )	6.54	n.d	7.138	19	9.82	n.d	Yes	
Western Canyon Lakes 1	4.85	0.719	7.53	11	14.6	6.53	Yes	Belilla et al. (2021)
Western Canyon Lakes 2	5.07	0.716	7.64	13.3	13.5	4.60	Yes	
Western Canyon Lakes 3	4.93	0.69	8.66	23.7	6.4	4.17	Yes	
Western Canyon Lakes 4	4.97	0.726	6.60	8.2	14.7	3.90	Yes	
Western Canyon Lakes 5	5.1	0.725	6.12	8.7	13.3	3.76	Yes	

*Labelled samples are abundant in chloride of iron, and their ISF was added to the ISF of MgCl_2_ when calculating the total I of MgCl_2_ frac.

It is intriguing to see that cavern 3 and 4, given that they belong to the same cavern field, exhibit significantly different chemical compositions, reflecting local changes in salt stratigraphy, influencing microbial behaviour at a cavern field scale. This observation highlights the necessity of site/cavern-specific investigation. It is evident that results cannot be extrapolated from one cavern to adjacent caverns, even within the same cavern field, as the salt stratigraphy might change locally, i.e. at a lateral scale of a few 100 m. Although mineralogical data from these sites are unavailable, future studies should investigate whether mineralogical fingerprinting could serve as a predictive tool for brine composition and/or chaotropicity, thereby informing microbial risk assessments and storage suitability.

To further test the robustness of the predictive model, published data from hypersaline environmental samples were used in our developed predictive model. Selected published studies from Payler et al. (2019), Moors et al. (2023), and Belilla et al. (2019, 2021^10–13^ were reprocessed to align with our interpretive framework. This involved converting reported energy-based chaotropicity values into relative agar gelling-point temperature changes and adjusting the sign of chaotropic and kosmotropic effects (see Sect.  3.1). The environmental brine predictions were evaluated using data from Payler et al. (2019) (Table 5), which included five brine samples from the Boulby Mine, UK (Billingham, 215, 29XC, 44XC, and 101-P). Microbial activity was tested using both real and synthetic brines with added carbon sources and inocula. Rapid growth occurred in Billingham, 215, and 44XC, while 29XC and 101-P showed no activity initially. Secondary testing led to growth in 29XC but not in 101-P. We predicted chaotropicity based on ionic composition and compared it to measured agar gelling-point temperatures. Both approaches identified Billingham, 215, and 44XC as kosmotropic, and 101-P as chaotropic. However, 29XC showed a difference: its measured gelling-point temperature (+ 22 °C) suggested kosmotropic behavior, while the predicted value (0 °C) was at the limit between chaotropicity and kosmotropicity. These findings suggest that our predictive model provides a better reflection of the environmental limits for microbial survival than relying solely on measured chaotropicity levels in brines.

The next set of environmental sample predictions was based on data from Moors et al. (2023) and Belilla et al. (2019, 2021), which investigated brines from the Danakil Depression in Ethiopia. These studies assessed the potential for life in poly-extreme environments and evaluated brine chaotropicity using agar gelling assays and microbiological analyses. Belilla et al. (2019, 2021) measured and calculated gelling temperatures, while Moors et al. (2023) relied on estimates based on Cray et al. (2013). (2013, 2015). We applied our predictive model to estimate agar gelling-point temperatures and assess chaotropicity for these brines (Table 5). Despite differences in magnitude, our predictions aligned with the chaotropicity/kosmotropicity classifications reported by Moors et al. and Belilla et al. Both Moors et al. (2023) and Belilla et al. (2019) detected active microbial life in samples from Lake Asale and Karum, consistent with their kosmotropic nature, an aspect better captured by our model than by direct measurements or indirect calculations. Belilla et al. (2021) also found thriving archaeal communities, dominated by Halobacteriota and Nanohaloarchaeota, in Western Canyon (WC) Lakes 1–4, which were similarly kosmotropic and well predicted by both our model and experimental measurements. These findings underline that high salinity alone does not limit life when pH and temperature remain within tolerable ranges^9^. Instead, brine composition plays a more critical role—specifically, the solution’s ionic strength and the proportion of chaotropic ions. These factors can be assessed through ion analysis, which enables the calculation of agar gelling-point temperature and the classification of brines as either chaotropic or kosmotropic. While agar gelling assays offer qualitative insights into brine chaotropicity, brine composition data are essential for guiding mitigation strategies of the microbes. Therefore, predictive models should incorporate mixed salt systems with more than two components to more accurately represent the complexity of environmental brines.

## Implications for H₂ storage in salt caverns

Microorganisms can thrive in high-salinity subsurface environments such as salt caverns^30^. Hence, it is crucial to monitor their activity during H₂ storage in these caverns due to the potential risks of H₂ loss, contamination, and cavern souring. As traditional biocides can have limited efficacy under high-salt conditions it is necessary to seek alternative methods to control or eliminate microbial activity. Natural brine can exhibit chaotropic characteristics, which can limit microbial growth, depending on additional factors including ionic strength, brine composition, pH, temperature and pressure, and nutrients. This suggests a novel strategy for potentially lowering the microbial risks at different stages of salt cavern development using chaotropic indigenous salts. At an early stage of exploration and geological screening for suitable areas for salt caverns, we propose to select areas where chaotropic salts are enriched, specifically magnesium- and calcium salt minerals. Secondly, during the development of new salt caverns or repurposing of existing salt caverns for H₂ storage, the analysis of brine composition can be used as an indicator of whether the cavern is at higher risk (being kosmotropic) or at lower risk (being chaotropic) for microbial activity. A next step could be the introduction of chaotropic salt solutions into the cavern sump to render the cavern chaotropic and minimize microbial activity, either as a pre-caution or as mitigation measure should adverse microbial activity be observed. If these mitigation options are indeed viable, they need to be assessed in future studies, also taking advantage of our developed method of measuring chaotropicity via agar gelling temperature assay or using the prediction model. This knowledge needs to be combined with microbiological growth and activity tests to further understand the effect of different chaotropic salts on microbial life. As chaotropicity effects and potential adaptations of microbes itself is not well studied, it can very well be that certain microbes can develop resistance, stabilizing membranes and protein structures, making activity possible even at high chaotropic values.

## Conclusion

This study investigated the chaotropic and kosmotropic characteristics of individual salts, salt mixtures, and four distinct salt cavern brines by measuring agar gelling temperatures using a newly developed oscillatory rheology method. The results showed that salts such as NaCl, KCl, CaCl₂, Na₂SO₄, MgSO₄, and Na₃Citrate exhibit kosmotropic behaviour, while MgCl₂ demonstrates chaotropicity based on its effect on agar gelling temperature. A predictive model was developed to define the chaotropic limits of brines in the presence of kosmotropic solutes, using binary mixtures of NaCl and MgCl₂. The model suggests that chaotropic behaviour occurs when the total ionic strength exceeds 3 mol/L, with at least 55% contributed by MgCl₂, or when it exceeds 6 mol/L, with 40% MgCl₂.

Furthermore, we predicted and measured the agar gelling temperature of four salt cavern brines. Caverns 1–3 exhibited kosmotropic characteristics, while cavern 4 was identified as chaotropic, indicating a lower microbial risk. This assessment was confirmed through cell number enumeration and microbial testing, which strongly support that cavern 4 can be considered a low-risk cavern for H₂ storage. We further challenged the predictive model with hypersaline environmental samples, in which the assignment of chaotropic/kosmotropic characteristics remined consistent same, despite observed differences in magnitude. Our findings confirm that high salinity alone does not inhibit life when pH and temperature are within optimal ranges. Instead, the total ionic strength and the proportion of chaotropic ions are more critical. This should be assessed through ion analysis to predict agar gelling temperature and thereby identify chaotropic or kosmotropic characteristics.

Based on these insights, we outlined a strategy to reduce microbial risks in salt caverns by adjusting brine composition to induce chaotropicity. While these predictions offer a promising step toward understanding and managing microbial activity in salt caverns, further microbiological studies are needed to validate the proposed mitigation strategies.

## Supplementary Information

Below is the link to the electronic supplementary material.

Supplementary Material 1

## Data Availability

The authors declare that the data supporting the findings of this study are available within the paper and its supplementary information files.
