# Supplementary material for: Predicting microbial activity potential in salt caverns based on brine chaotropicity analysis
Source: Sci Rep. 2026 Feb 23;16:10235. doi: 10.1038/s41598-026-40866-z (PMC13031867; doi:10.1038/s41598-026-40866-z)
Supplement: Supplementary file 1 — Supplementary Material 1 [file 41598_2026_40866_MOESM1_ESM.docx]

**SUPPLEMENTAL INFORMATION**

**Predicting microbial activity potential in salt caverns based on brine chaotropicity analysis**

Abduljelil Kedir^1^, Kyle Mayers^1^, Janiche Beeder^2^, Silvan Hoth^2^, Nicole Dopffel^1*^

^1^ NORCE Norwegian Research Center AS, Norway

^2^ Equinor ASA, Norway

*Corresponding author: nicd@norceresearch.no


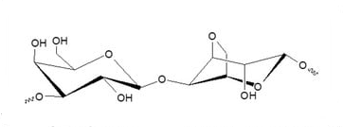

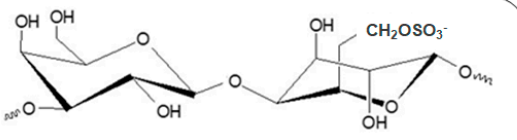


**Agarose**

**Agaropectin**

Supplementary Figure 1: Chemical structure of agar.


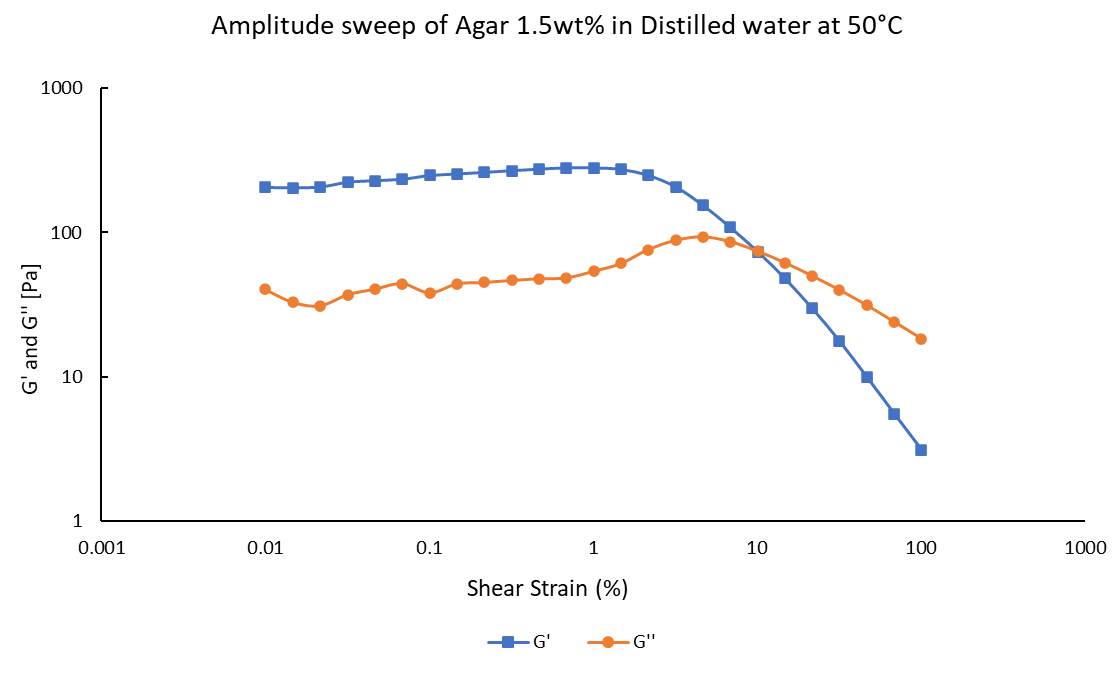


Supplementary Figure 2: The amplitude sweep measurement of 1.5 wt% in distilled water at 50°C


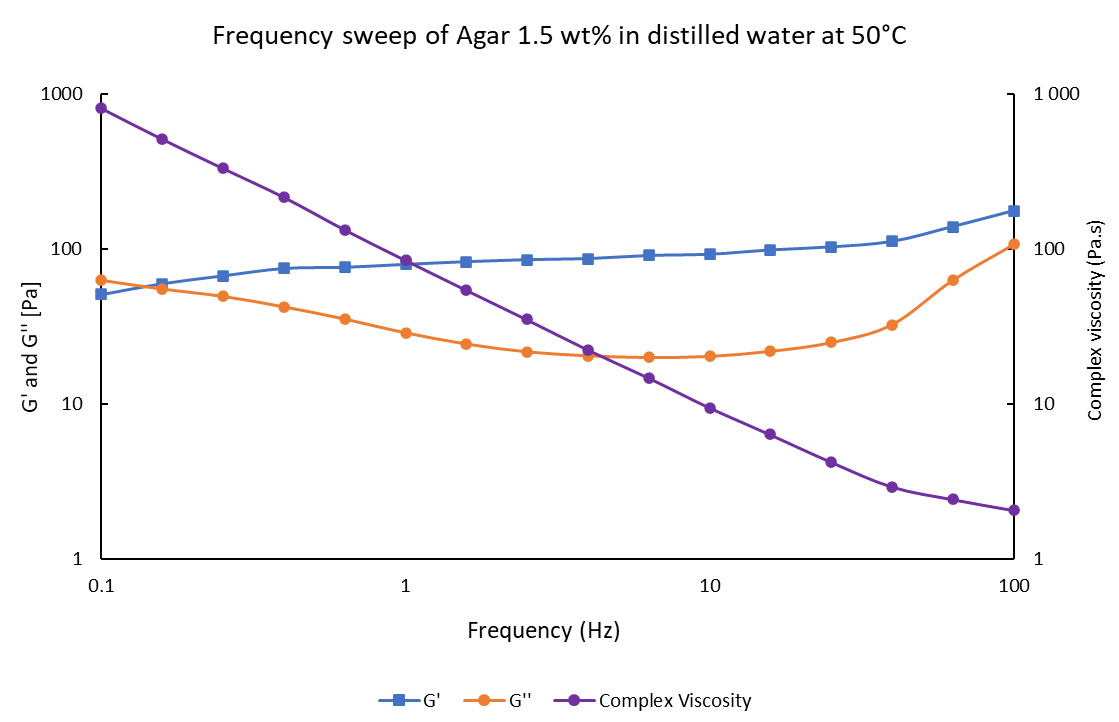
Supplementary Figure 3: The frequency sweep measurement of 1.5 wt% in distilled water at 50°C


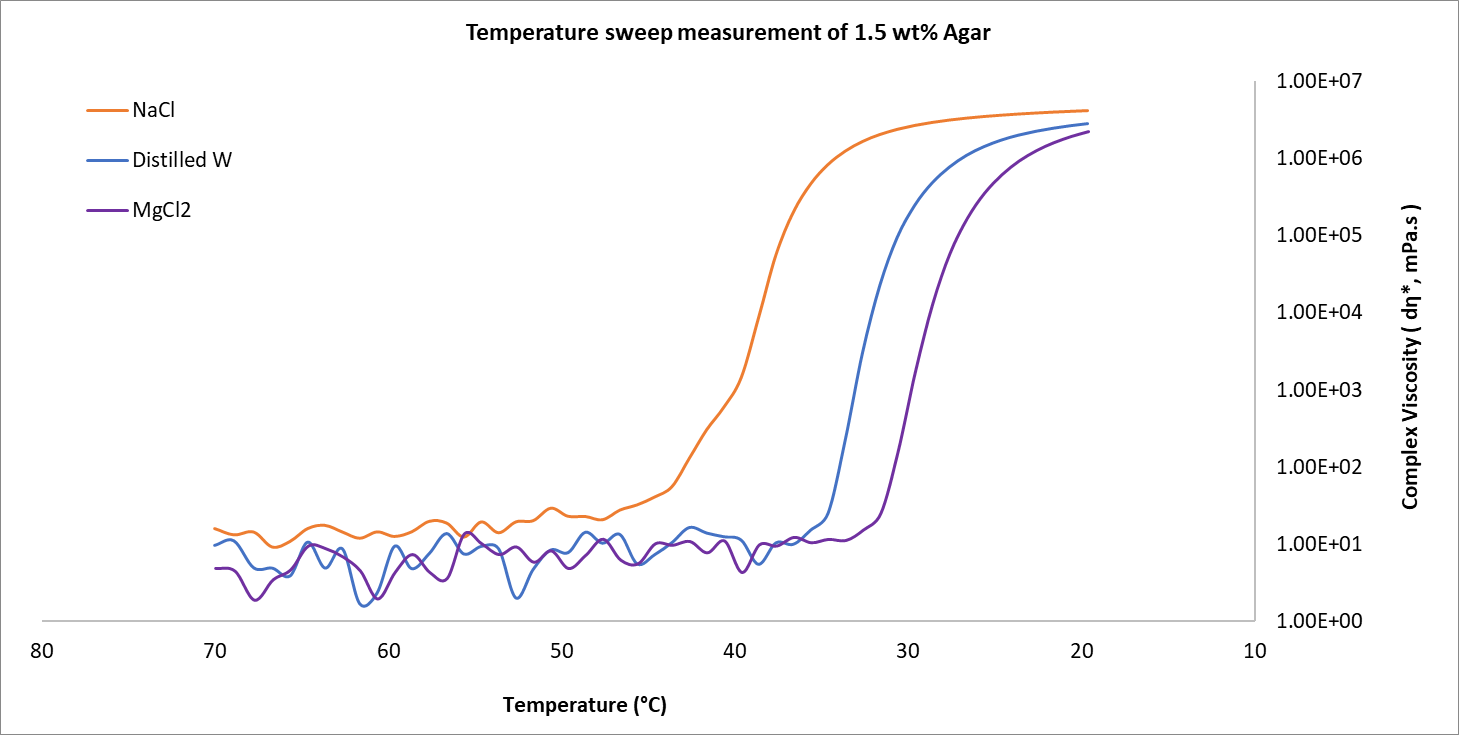


Supplementary Figure 4: A temperature sweep measurement of 1.5% agar in distilled water, NaCl (ionic strength of 2.18 mol/L), and MgCl_2_ (ionic strength of 2.18 mol/L)

Supplementary Table S1. The concentration and the corresponding ionic strength of binary salt mixtures of NaCl and MgCl_2_ with varying ionic strength fractions (ISF) of MgCl_2_ while maintaining the total ionic strength (TIS) at 4.10 mol/L.

| [NaCl] (mol/L) | [MgCl_2_^.^6H_2_O] (mol/L) | IS of NaCl | IS of MgCl_2_^.^6H_2_O | ISF of MgCl2 (%) | TIS (mol/L) |
| --- | --- | --- | --- | --- | --- |
| 4.10 | 0.00 | 4.10 | 0.00 | 0 % | 4.10 |
| 3.69 | 0.14 | 3.69 | 0.41 | 10 % | 4.10 |
| 3.08 | 0.34 | 3.08 | 1.03 | 25 % | 4.10 |
| 2.46 | 0.55 | 2.46 | 1.64 | 40 % | 4.10 |
| 1.85 | 0.75 | 1.85 | 2.26 | 55 % | 4.10 |
| 1.23 | 0.96 | 1.23 | 2.87 | 70 % | 4.10 |
| 0.62 | 1.16 | 0.62 | 3.49 | 85 % | 4.10 |
| 0.00 | 1.37 | 0.00 | 4.10 | 100 % | 4.10 |

*
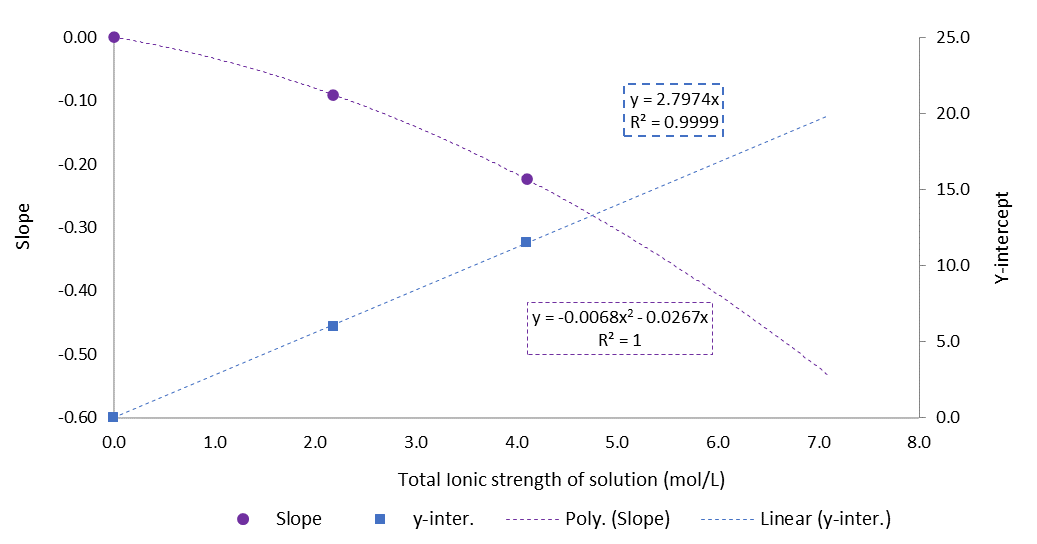
*

Supplementary Figure 5. The slope and y-intercept of 1.5% agar gelling point temperature in a mixture of NaCl and MgCl_2_ as a function of the total ionic strength of the solution (TIS).

|  | Salt Cavern 1 (g/L) | Salt Cavern 2 (g/L) | Salt Cavern 3 (g/L) | Salt Cavern 4 (g/L) |
| --- | --- | --- | --- | --- |
| Na^+^ | 124.59 | 127.75 | 113.34 | 46.43 |
| K^+^ | 0.46 | 0.07 | 9.27 | 33.45 |
| Ca^2+^ | 1.02 | 1.36 | 0.37 | 0.19 |
| Mg^2+^ | 1.03 | 0.07 | 7.31 | 34.50 |
| Cl^-^ | 198.47 | 192.66 | 194.20 | 200.98 |
| SO_4_^2-^ | 4.46 | 2.85 | 12.78 | 19.58 |
| HCO_3_^-^ | 0.10 | 0.03 | _ | _ |

Supplementary Table S2. The brine composition of the four salt caverns


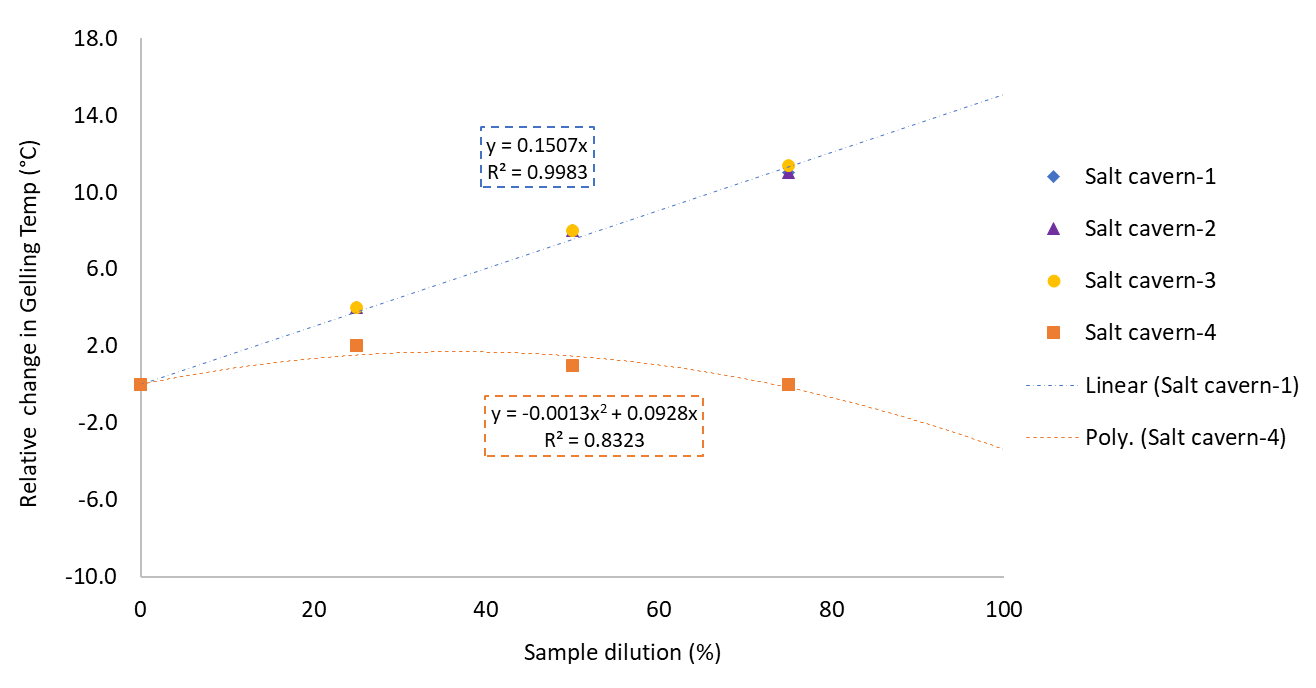


Supplementary Figure 6: The relative change in agar gelling point temperature as a function of the percent of sample dilution for salt cavern samples of 1-4.

Supplementary Table S3: Fermentation activity in cavern 1 -4. All brine samples were amended with 10 mM glucose and 0.01 yeast extract and incubated at 30°C.

| Cavern 1 |  | 0 | 56 |  |  |
| --- | --- | --- | --- | --- | --- |
|  | Acetate [mg/L] | 0 | 136 |  |  |
|  | Glucose [mg/L] | 1600 | 1153 |  |  |
|  | Hydrogen formation | | yes |  |  |
|  |  |  |  |  |  |
| Cavern 2 |  | 0 | 30 |  |  |
|  | Acetate [mg/L] | 0 | 0 |  |  |
|  | Glucose [mg/L] | 1801 | 766.4 |  |  |
|  | Hydrogen formation | | yes |  |  |
|  |  |  |  |  |  |
| Cavern 3 |  | 0 | 172 | 223 | 307 |
|  | Acetate [mg/L] | 8.1 | 11.2 | 8.7 | 20.0 |
|  | Glucose [mg/L] | 1600.4 | 1520.5 | 1550.0 | 1506.2 |
|  | Hydrogen formation | | no | yes | yes |
|  |  |  |  |  |  |
| Cavern 4 |  | 0 | 100 | 159 | 208 |
|  | Acetate [mg/L] | 0 | 0 | 0 | 0 |
|  | Glucose [mg/L] | 1398.2 | 1250.9 | 1294.9 | 1353.8 |
|  | Hydrogen formation | | no | no | no |

Supplementary Table S4-7: Hydrogen loss over time in mmol and H_2_S in ppm in the headspace and pH measured in pure salt cavern brines (cavern 1 – 4) with 100% hydrogen in the headspace incubated at 30°C. For each cavern distilled water controls with 100% hydrogen in the headspace are run in parallel to measure abiotic hydrogen loss.

|  | 0 | 56 | 96 | 136 | 176 |
| --- | --- | --- | --- | --- | --- |
| Cavern 1 - H_2_ | 1.90 | 1.83 | 1.71 | 1.67 | 1.64 |
| Cavern 1 - H_2_ | 1.92 | 1.86 | 1.74 | 1.68 | 1.66 |
| Control 1 – H_2_ | 2.44 | 2.43 | 2.42 | 2.41 | 2.37 |
| Cavern 1 – H_2_S | 0 | - | 763 | 1450 | 780 |
| Cavern 1 - H_2_S | 0 | - | 882 | 1561 | 977 |
| Cavern 1 – pH | 7.5 | 7.9 | 8.4 | 8.5 | 8.6 |
| Cavern 1 - pH |  | 7.9 | 8.4 | 8.4 | 8.1 |

|  | 0 | 71 | 116 | 162 | 252 | 363 |
| --- | --- | --- | --- | --- | --- | --- |
| Cavern 2 - H_2_ | 1.77 | 1.64 | 1.64 | 1.55 | 1.52 | 1.39 |
| Cavern 2 - H_2_ | 1.81 | 1.67 | 1.67 | 1.54 | 1.46 | 1.36 |
| Control 2 – H_2_ | 2.08 | 1.95 | 1.97 | 1.93 | - | - |
| Control 2 – H_2_ | 2.16 | 2.05 | 2.02 | 1.98 | - | - |
| Cavern 2 – H_2_S | 0 | 0 | 0 | 0 | 0 | 813 |
| Cavern 2 - H_2_S | 0 | 0 | 0 | 910 | 1857 | 1589 |
| Cavern 2 – pH | 6.9 | 6.6 | - | 6.7 | 6.5 | 7.5 |
| Cavern 2 - pH | 6.9 | 6.9 | - | 7.5 | 7.5 | 8.1 |

|  | 0 | 54 | 91 | 145 | 200 | 264 | 312 |
| --- | --- | --- | --- | --- | --- | --- | --- |
| Cavern 3 - H_2_ | 2.15 | 2.14 | 2.12 | 2.12 | 2.08 | 2.08 | 1.95 |
| Cavern 3 - H_2_ | 2.12 | 2.13 | 2.12 | 2.10 | 2.05 | 2.04 | 1.96 |
| Control 3 – H_2_ | 1.69 | 1.69 | 1.72 | 1.71 | 1.69 | 1.66 | - |
| Control 3 – H_2_ | 1.82 | 1.81 | 1.81 | 1.80 | 1.78 | 1.75 | 1.69 |
| Cavern 3 – H_2_S | 0 | 0 | 0 | 0 | 0 | 0 | 0 |
| Cavern 3 - H_2_S | 0 | 0 | 0 | 0 | 0 | 0 | 0 |
| Cavern 3 – pH | 6.5 | 6.6 | 6.7 |  | 6.6 | 6.8 | 6.3 |
| Cavern 3 - pH | 6.6 | 6.6 | 6.7 |  | 6.7 | 6.8 | 6 |

|  | 0 | 50 | 103 | 131 | 159 | 208 | 256 | 403 |
| --- | --- | --- | --- | --- | --- | --- | --- | --- |
| Cavern 4 - H_2_ | 2.27 | 1.95 | 1.93 | 1.78 | 1.84 | 1.72 | 1.71 | 1.62 |
| Cavern 4 - H_2_ | 2.22 | 1.90 | 1.88 | 1.77 | 1.83 | 1.71 | 1.72 | 1.65 |
| Control 4 – H_2_ | 2.25 | 1.93 | 1.90 | 1.80 | 1.83 | 1.71 | 1.69 | 1.65 |
| Control 4 – H_2_ | 2.18 | 1.88 | 1.85 | 1.75 | 1.78 | 1.63 | 1.64 | 1.57 |
| Cavern 4 – H_2_S | 0 | 0 | 0 | 0 | 0 | 0 | 0 | 0 |
| Cavern 4 - H_2_S | 0 | 0 | 0 | 0 | 0 | 0 | 0 | 0 |
| Cavern 4 – pH | 6 | 6.1 | 6 | 5.6 | - | 6 | 6.1 | 5.7 |
| Cavern 4 - pH | 6 | 6 | 5.7 | 5.5 | - | 6.1 | 6 | 5.7 |
